# Safety and tolerability of donepezil 23 mg in moderate to severe Alzheimer's disease

**DOI:** 10.1186/1471-2377-11-57

**Published:** 2011-05-25

**Authors:** Martin Farlow, Felix Veloso, Margaret Moline, Jane Yardley, Elimor Brand-Schieber, Francesco Bibbiani, Heng Zou, Timothy Hsu, Andrew Satlin

**Affiliations:** 1 Department of Neurology, Indiana University School of Medicine, 541 Clinical Drive, CL299, Indianapolis, IN, 46202, USA; 2 Centre for Clinical Cognitive Research, Pasqua Hospital, 4101 Dewdney Avenue, Regina, Saskatchewan, S4T 1A5, Canada; 3 Eisai Inc, Neuroscience Product Creation Unit, 100 Tice Blvd., Woodcliff Lake, NJ 07677, USA; 4 Eisai Ltd, Neuroscience, Mosquito Way, Hatfield, Hertfordshire, AL10 9SN, UK

## Abstract

**Background:**

Donepezil 23 mg/d, recently approved in the United States for treatment of moderate to severe Alzheimer's disease (AD), was developed to address the need for an additional treatment option for patients with advanced AD. This report, based on a pivotal phase 3 study, presents a detailed analysis of the safety and tolerability of increasing donepezil to 23 mg/d compared with continuing 10 mg/d.

**Method:**

Safety analyses comprised examination of the incidence, severity, and timing of treatment-emergent adverse events (AEs) and their relationship to treatment initiation; changes in weight, electrocardiogram, vital signs, and laboratory parameters; and the incidence of premature study discontinuation. The analysis population (n = 1434) included all randomized patients who took at least 1 dose of study drug and had a postbaseline safety assessment. To further examine the effect of transition from a lower to a higher donepezil dose, a pooled analysis of safety data from 2 phase 3 trials of donepezil 5 mg/d and 10 mg/d was also performed.

**Results:**

The safety population comprised 1434 patients: donepezil 23 mg/d (n = 963); donepezil 10 mg/d (n = 471); completion rates were 71.1% and 84.7%, respectively. The most common AEs were nausea, vomiting, and diarrhea (donepezil 23 mg/d: 11.8%, 9.2%, 8.3%; donepezil 10 mg/d: 3.4%, 2.5%, 5.3%, respectively). AEs that contributed most to early discontinuations were vomiting (2.9% of patients in the 23 mg/d group and 0.4% in the 10 mg/d group), nausea (1.9% and 0.4%), diarrhea (1.7% and 0.4%), and dizziness (1.1% and 0.0%). The percentages of patients with AEs in the 23 mg/d group, as well as the timing, type, and severity of these AEs, were similar to those seen in previous donepezil trials with titration from 5 to 10 mg/d. Serious AEs were uncommon (23 mg/d, 8.3%; 10 mg/d, 9.6%).

**Discussion:**

The 23 mg/d dose of donepezil was associated with typical cholinergic AEs, particularly gastrointestinal-related AEs, similar to those observed in studies with a dose increase from 5 to 10 mg/d.

**Conclusion:**

The good safety and predictable tolerability profile for donepezil 23 mg/d supports its favorable risk/benefit ratio in patients with moderate to severe AD.

**Trial Registration:**

NCT00478205

## Background

Efficacy and safety of once-daily donepezil 23 mg, an acetylcholinesterase inhibitor (AChEI), were investigated in a recent clinical trial involving 1467 randomized patients with moderate to severe Alzheimer's disease (AD) (NCT00478205) [1]. Compared with patients who continued taking donepezil 10 mg/d, those whose dose was increased to donepezil 23 mg/d demonstrated a significantly greater cognitive benefit on the Severe Impairment Battery (SIB) after 6 months of treatment.

AChEIs as a class may cause cholinergic adverse events (AEs). The most frequently occurring treatment-related AEs are nausea, vomiting, and diarrhea, and the frequency and severity of these effects are related to dose [2,3]. Less common class-related AEs include vagotonic (eg, bradycardia, syncope), central nervous system (eg, aggression, sleep disturbance), and parasympathetically mediated (eg, urinary incontinence) AEs [4,5]. Gastrointestinal (GI) AEs typically increase after dose initiation or upward dose titration, after which the frequency declines within a few weeks [3,4].

This report is a detailed presentation and analysis of the safety and tolerability data from the previously published clinical trial [1] comparing an increase in the daily dose of donepezil from 10 mg/d to 23 mg/d with continuation on 10 mg/d. These analyses substantively extend the presentation of safety and tolerability findings previously published. In addition, this paper includes an analysis of pooled data from 2 previous double-blind, placebo-controlled, 24-week trials in which donepezil was initiated at 5 mg/d and titrated to 10 mg/d [6,7] in order to further clarify the relationship between dose transitions and AEs. The primary objective is to help guide clinicians who are treating patients with moderate to severe AD at this new dosage.

## Methods

### Objectives and study design

The protocol and informed-consent form were approved by the independent ethics committee/institutional review board at each independent research site and conformed to the principles of the World Medical Association's Declaration of Helsinki and all local regulations. The study design was reviewed and deemed appropriate by the US Food and Drug Administration (FDA) and other global regulatory agencies.

The primary objective of the study reported by Farlow et. al. [1] was to compare the efficacy and safety of escalation to the higher donepezil dose (23 mg/d) versus continuation on the 10 mg/d dose. The design and methods of this study have been described previously [1]. Briefly, this randomized, double-blind, parallel-group study included patients with moderate to severe AD (Mini-Mental State Examination [MMSE] score 0-20 at baseline) who had already been taking donepezil 10 mg/d for at least 3 months. Patients who were taking memantine at doses of ≤ 20 mg/d for at least 3 months prior to screening could enroll, provided they agreed to maintain that dosage throughout the trial. Patients were randomized 2:1 to either increase to donepezil 23 mg/d or continue donepezil 10 mg/d for 24 weeks. Randomization was stratified by memantine use at baseline.

### Safety analysis

Safety analyses comprised examination of the incidence, severity, and timing of treatment-emergent AEs and their relationship to treatment initiation; changes in weight, electrocardiogram (ECG), vital signs, and laboratory parameters; and the incidence of premature discontinuation from the study. Serious AEs were defined as those that resulted in death or were life-threatening or required hospitalization or prolongation of hospitalization, or created persistent or significant disability/incapacity. Safety and tolerability were assessed in the safety population (n = 1434), which included all randomized patients who took at least 1 dose of study drug and had at least 1 postbaseline safety assessment. In addition to the planned analyses described above, post hoc analyses were conducted to examine safety and tolerability based on intrinsic and extrinsic patient characteristics, including demographic variables. Age categories were selected to comply with regulatory reporting guidelines and are limited at the low and high ends by study entry criteria. Weight groupings were selected to provide both consistent ranges and comparable numbers of subjects. The impact of concomitant memantine use on AEs was also examined.

### Analysis of pooled data from prior studies

To further compare the impact of titration from a lower to a higher donepezil dose, a pooled analysis was performed on previously published data from studies of patients with mild to moderate AD newly receiving donepezil [6,7]. All patients in both of these studies received 5 mg/d or placebo during Week 1; from Week 2 until the end of the study, groups received placebo, donepezil 5 mg/d, or donepezil 10 mg/d. The forced dose titration took place on study Day 8. Data were analyzed for cumulative percentage of patients with AEs.

## Results

### Demographics and baseline characteristics

The baseline demographic profile of patients included in the safety population of the study by Farlow et al. is shown in Table 1. All baseline values were similar between the 2 treatment groups. Prior to baseline, patients in both treatment groups had been taking donepezil for a mean (± SD) of 110 (105) weeks (range, 10-606 weeks).

**Table 1:** Baseline demographic profile of patients in the safety population

	Donepezil 23 mg/d (n = 963)	Donepezil 10 mg/d (n = 471)
Age (years), mean (SD)	73.9 (8.53)	73.8 (8.56)
Gender, n (%)		
Male	356 (37.0)	177 (37.6)
Female	607 (63.0)	294 (62.4)
Race, n (%)		
White	708 (73.5)	346 (73.5)
Asian/Pacific	161 (16.7)	87 (18.5)
Hispanic	67 (7.0)	26 (5.5)
Black	22 (2.3)	9 (1.9)
Other	5 (0.5)	3 (0.6)
Weight (kg)		
<55	218 (22.6)	111 (23.6)
≥55	744 (77.3)	360 (76.4)
Missing	1 (0.1)	0 (0.0)
Concomitant memantine use, n (%)		
Memantine	352 (36.6)	168 (35.7)
None	611 (63.4)	303 (64.3)
Prior donepezil use (weeks)		
Mean (SD)	112.2 (108.2)	104.8 (99.0)
Range	9.9-574.6	10.4-606.4
Baseline MMSE		
Mean (SD)	13.1 (4.99)	13.0 (4.75)

### Patient disposition

**Figure 1:**
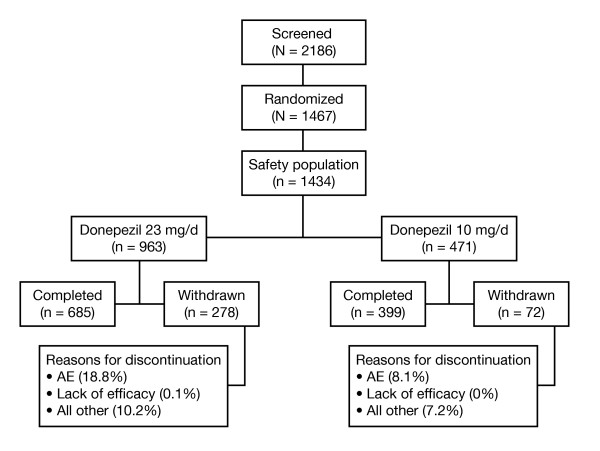
**Patient disposition**.

Of 1467 patients in the 10 mg/d vs 23 mg/d study who were randomized to treatment, 981 (66.9%) received donepezil 23 mg/d and 486 (33.1%) received donepezil 10 mg/d. The safety population comprised 1434 patients: 963 in the donepezil 23 mg/d group and 471 in the donepezil 10 mg/d group (Figure 1). Sixteen patients (9 donepezil 23 mg/d and 7 donepezil 10 mg/d) discontinued from the study prior to receiving the study drug and 17 patients (9 donepezil 23 mg/d and 8 donepezil 10 mg/d) received at least 1 dose of study medication but were not included in the safety population because of lack of a postbaseline safety assessment. Completion rates for the patients in the safety population were 71.1% and 84.7% in the 23 mg/d and 10 mg/d treatment groups, respectively. AEs were the most common reason for discontinuation (23 mg/d = 18.8%, 10 mg/d = 7.9%); the AEs that contributed to the greatest number of early discontinuations were vomiting, nausea, diarrhea, and dizziness (Table 2).

**Table 2:** AEs leading to discontinuation that occurred in >1.0% of patients in the 23 mg/d treatment group

Preferred Term	Donepezil 23 mg/d, n (%) (n = 963)	Donepezil 10 mg/d, n (%) (n = 471)
**Total**	179 (18.6)	37 (7.9)
Vomiting	28 (2.9)	2 (0.4)
Nausea	18 (1.9)	2 (0.4)
Diarrhea	16 (1.7)	2 (0.4)
Dizziness	11 (1.1)	0 (0.0)

### Overall safety and tolerability

#### Overview

As has been reported previously [1], AEs were generally more common in the 23 mg/d group, occurring in 73.7% of patients, compared with 63.7% of patients in the donepezil 10 mg/d group (Table 3). Of patients who experienced AEs, the majority (88.6% in the 23 mg/d group and 88.7% in the 10 mg/d group) had AEs of mild to moderate severity. The most common AEs--the only AEs that occurred in >5% of patients in either group--were nausea, vomiting, diarrhea, and anorexia.

**Table 3:** AEs that occurred in ≥ 2% of patients in the donepezil 23 mg/d group and at higher frequency than in the donepezil 10 mg/d group

Preferred term	Donepezil 23 mg/d (n = 963)	Donepezil 10 mg/d (n = 471)
Percentage of patients with at least 1 AE	73.7	63.7
Nausea	11.8	3.4
Vomiting	9.2	2.5
Diarrhea	8.3	5.3
Anorexia	5.3	1.7
Dizziness	4.9	3.4
Weight decreased	4.7	2.5
Urinary tract infection	4.4	4.0
Headache	4.3	3.2
Fall	4.0	3.8
Agitation	3.9	3.8
Insomnia	3.4	2.3
Bradycardia	2.7	0.6
Sinus bradycardia	1.1	0.0
Aggression	2.7	2.5
Urinary incontinence	2.5	1.3
Fatigue	2.4	0.8
Asthenia	2.1	0.6
Contusion	2.1	0.2
Somnolence	2.1	1.1

Overall, 125 (8.7%) patients experienced at least 1 serious AE during the study (Table 4). The incidence of serious AEs was similar in the 23 mg/d group (8.3%) and the 10 mg/d group (9.6%). Serious AEs that were deemed possibly or probably related to study drug were also similar in frequency in the 23 mg/d group (2.8%) and the 10 mg/d group (2.3%). There were 13 deaths during the study or within 30 days of the study end: 8 (0.8%) in the donepezil 23 mg/d group and 5 (1.1%) in the 10 mg/d group. None of these deaths was considered related to the study medication.

**Table 4:** Serious AEs that occurred in >0.5% of patients in either treatment group

Preferred Term	Donepezil 23 mg/d (n = 963)	Donepezil 10 mg/d (n = 471)
Percentage of patients with at least 1 serious AE	8.3	9.6
Urinary tract infection	0.6	0.4
Fall	0.6	0.4
Pneumonia	0.3	0.6
Syncope	0.2	1.1
Aggression	0.2	0.8
Confusional state	0.1	0.6

#### AEs of interest

Of particular interest are those AEs that have potentially important clinical consequences. In the present analysis, for this large cohort of patients with moderate to severe AD, this includes those AEs commonly associated with more advanced AD, such as falls and psychiatric symptoms, and those that are cholinergic-related, including GI bleeding, bradycardia, and weight loss.

##### GI bleeding

There was a higher incidence of GI bleeding (in total, including the following terms: GI hemorrhage, hematemesis, hematochezia, hemorrhagic diarrhea, melena, and rectal bleeding) in the 23 mg/d group (1.1%) than in the 10 mg/d group (0.6%). Of the 14 GI bleeding events, all 7 serious cases occurred in the 23 mg/d group. Of these, 3 were considered possibly related to study medication; in 2 of these cases the patient was discontinued from the study.

##### Bradycardia

There were 27 (2.8%) cases of bradycardia in the 23 mg/d group (including 11 cases of sinus bradycardia, which is a distinct term in the Medical Dictionary for Regulatory Activities), compared with 3 (0.6%) cases in the 10 mg group. All but 1 of the bradycardia events in the 23 mg/d group were mild or moderate in severity; none of the events in the 10 mg/d group was severe and only 4 of the 23 mg/d events were defined as serious. Of the 4 patients who had a serious event, 2 stayed on drug and completed the study, 2 discontinued the study; all had resolution of their bradycardia. In the month preceding or following the bradycardia event, 2 cases were associated with dizziness, 1 with a fall, and none with syncope. Eight patients in the 23 mg/d group and no patients in the 10 mg/d group discontinued from the study because of bradycardia.

##### Weight loss

Weight loss was reported as an AE more frequently in the donepezil 23 mg/d group (4.7%) than in the donepezil 10 mg/d group (2.5%). By the end of the study, 8.4% of patients in the donepezil 23 mg/d group experienced a weight decrease of ≥ 7%, compared with 4.9% of those in the 10 mg/d group.

##### Falls

The percentage of patients experiencing falls was similar in the 23 mg/d and 10 mg/d groups (4.0% vs 3.8%). Twenty-nine of 39 cases (74.4%) in the 23 mg/d group and 13 of 18 cases (72.2%) in the 10 mg/d group were not considered related to study medication.

##### Psychiatric symptoms

In the 2 treatment groups, there were similar rates of agitation (3.9% in the 23 mg/d group vs 3.8% in the 10 mg/d group), aggression (2.7% vs 2.5%), and hallucinations (1.1% vs 0.8%).

#### Treatment-emergent laboratory abnormalities, ECG, or vital signs

No clinically important differences between treatment groups were observed in the proportion of patients with treatment-emergent abnormal laboratory values or ECG abnormalities other than bradycardia, or in the magnitude or direction of mean change from baseline for any vital sign parameter.

### Demographic and baseline factors: effect on safety and tolerability

#### Age

For analysis of the effects of age, the following age groups were defined: 45-64 years (n = 235), 65-74 years (n = 440), 75-84 years (n = 643), and 85-90 (n = 116) years. In both treatment groups, there was a higher rate of diarrhea and urinary tract infection with increasing age; in the 23 mg/d treatment group, but not in the 10 mg/d group, fatigue, somnolence, and urinary incontinence also demonstrated this tendency. Interestingly, the rate of nausea and vomiting generally tended to decrease in successively higher age groups among those receiving 23 mg/d, from 15.6% and 10.5%, respectively, in the youngest age group to 6.5% and 2.6% in the highest.

#### Gender

There were few notable gender differences. There was a similar high rate of urinary tract infection in women in both treatment groups (5.9% for 23 mg/d and 5.4% for 10 mg/d) compared with 1.7% in men in both groups. In the 23 mg/d treatment group specifically, the only AEs that were noted more frequently in women than in men were anorexia (6.3% vs 3.7%, respectively) and weight decrease (5.8% vs 2.8%, respectively). In contrast, there was a slightly higher incidence in men than in women of agitation in the 23 mg/d group (5.9% vs 2.8%) and of aggression in both treatment groups (3.4% vs 2.3% for 23 mg/d and 4.0% vs 1.7% for 10 mg/d).

#### Race

Because there were too few black and Hispanic patients to provide meaningful conclusions, comparisons could be made only between white patients (73.5% of patients in this analysis) and Asian/Pacific patients (17.3% of patients in this analysis). In both treatment groups, there were higher incidences of nausea, vomiting, anorexia, dizziness, headache, and insomnia among Asian/Pacific patients than among white patients. This may be related to Asians having a lower mean baseline weight (55.1 kg) compared to the total safety population (66.3 kg) (see below).

#### Weight

In the donepezil 23 mg/d treatment arm, a higher incidence of AEs was observed in the lowest weight group (≤55 kg, n = 218) compared with each of the 3 higher weight groups (55 to <65, n = 245; 65 to <75, n = 240; ≥75, n = 259).

#### Concomitant memantine use

Similar types of AEs were observed in patients regardless of memantine use. The patients taking memantine tended to have more advanced AD at baseline (mean MMSE score among patients taking memantine was about 12 points in both treatment groups and about 14 in those not taking memantine). As expected, patients in both treatment groups who were also taking memantine concomitantly tended to have higher rates of those AEs that were commonly observed in patients with more severe disease, such as agitation and falls. Rates of serious AEs were also slightly higher in both treatment groups among patients using memantine.

### Timing of AEs and discontinuations

#### Higher dose study

As shown in Table 5, at the end of Week 1 there was a higher rate of patients reporting any AE and of patients discontinuing due to AEs in the 23 mg/d group compared with the group that continued taking 10 mg/d (30% and 8.3% vs 12.1% and 1.9%). However, the absolute increase in these percentages was similar in both groups between Weeks 1 and 2 and again between Weeks 2 and 4. Finally, there were comparable increases in the percentage of patients reporting any AE between Week 4 and end of study in the 23 mg/d and 10 mg/d groups.

**Table 5:** Cumulative percent of patients with AEs

	Donepezil 23 mg/d				Donepezil 10 mg/d			
	
	1 week	2 weeks	4 weeks	Total duration	1 week	2 weeks	4 weeks	Total duration
Number (%) of subjects with at least 1 such AE	289 (30.0)	347 (36.0)	444 (46.1)	710 (73.7)	57 (12.1)	83 (17.6)	124 (26.3)	300 (63.7)
Number (%) of subjects who discontinued due to AEs	80 (8.3)	98 (10.2)	122 (12.7)	179 (18.6)	9 (1.9)	16 (3.4)	20 (4.2)	37 (7.9)
Vomiting	68 (7.1)	73 (7.6)	77 (8.0)	89 (9.2)	5 (1.1)	5 (1.1)	5 (1.1)	12 (2.5)
Nausea	92 (9.6)	96 (10.0)	100 (10.4)	114 (11.8)	6 (1.3)	7 (1.5)	9 (1.9)	16 (3.4)
Diarrhea	34 (3.5)	44 (4.6)	57 (5.9)	80 (8.3)	8 (1.7)	11 (2.3)	12 (2.5)	25 (5.3)
Dizziness	26 (2.7)	28 (2.9)	35 (3.6)	47 (4.9)	3 (0.6)	6 (1.3)	8 (1.7)	16 (3.4)
Headache	18 (1.9)	22 (2.3)	30 (3.1)	41 (4.3)	1 (0.2)	2 (0.4)	5 (1.1)	15 (3.2)

#### Comparison with analysis of pooled data from prior studies

To further examine the effect of donepezil dose up-titration, rates of AEs associated with initiation of treatment with 5 mg/d donepezil and with a dose increase from 5 mg/d to 10 mg/d were assessed using pooled data from 2 pivotal phase 3 trials of donepezil for the treatment of mild to moderate AD [6,7]. Both active treatment groups were initiated on 5 mg/d at baseline. On study Day 8, the 5 mg group continued taking that dose, and the comparator group titrated up from 5 mg to 10 mg. During the first week after starting 5 mg, the rate of AEs overall and of discontinuations due to AEs was similar in the placebo and active treatment groups (Table 6). However, during Week 2, the percentages of patients with AEs and patients discontinuing due to AEs both increased markedly in the group that was titrated to 10 mg/d (absolute increase of 20.7% and 3.5%, respectively). Such marked increases were not observed in the 5 mg/d group, which had increases of 6.9% and 0.7%, respectively, comparable to the placebo group (6.5% and 0.7%, respectively). From the end of Week 2 to the end of Week 4, the absolute increase in the percentage of patients reporting AEs was once again similar among all groups (11.2%, 11.8%, and 10.5% in the placebo, 5 mg/d, and 10 mg/d groups, respectively), although the percentage of patients discontinuing due to AEs remained elevated in the 10 mg/d group (0.7%, 0.5%, and 3.2%, respectively). This pattern continued from Week 4 to the end of the study, just as observed for the 23 mg/d group (Table 4).

**Table 6:** Cumulative percent of patients with AEs (302/304 pooled population)

	Placebo				Donepezil 5 mg				Donepezil 10 mg			
	
	1 week	2 weeks	4 weeks	Total duration	1 week	2 weeks	4 weeks	Total duration	1 week*	2 weeks	4 weeks	Total duration
Number (%) of subjects with at least 1 such AE	92 (21.1)	122 (28.0)	171 (39.2)	329 (75.5)	98 (23.1)	126 (29.6)	176 (41.4)	331 (77.9)	118 (27.4)	201 (48.1)	252 (58.6)	361 (84.0)
Number (%) of subjects who discontinued due to AE	8 (1.8)	11 (2.5)	14 (3.2)	27 (6.2)	5 (1.2)	8 (1.9)	10 (2.4)	20 (4.7)	9 (2.1)	24 (5.6)	38 (8.8)	65 (15.1)
Vomiting	2 (0.5)	2 (0.5)	3 (0.7)	13 (3.0)	7 (1.6)	9 (2.1)	10 (2.4)	17 (4.0)	13 (3.0)	38 (8.8)	47 (10.9)	59 (13.7)
Nausea	6 (1.4)	11 (2.5)	14 (3.2)	25 (5.7)	15 (3.5)	16 (3.8)	19 (4.5)	26 (6.1)	22 (5.1)	62 (14.4)	78 (18.1)	92 (21.4)
Diarrhea	4 (0.9)	6 (1.4)	9 (2.1)	22 (5.0)	9 (2.1)	15 (3.5)	18 (4.2)	42 (9.9)	14 (3.3)	31 (7.2)	45 (10.5)	73 (17.0)
Dizziness	4 (0.9)	8 (1.8)	10 (2.3)	21 (4.8)	8 (1.9)	14 (3.3)	15 (3.5)	28 (6.6)	9 (2.1)	20 (4.7)	26 (6.0)	38 (8.8)
Headache	9 (2.1)	10 (2.3)	19 (4.4)	50 (11.5)	7 (1.6)	11 (2.6)	20 (4.7)	41 (9.6)	17 (4.0)	31 (7.2)	36 (8.4)	57 (13.3)

*Patients received 5 mg per day during Week 1 and titrated to 10 mg/d on the first day of study Week 2.

## Discussion

Both the 23 mg/d and 10 mg/d doses of donepezil used in the study reported by Farlow et al. were generally safe and well tolerated. A transient increase in cholinergic-related GI AEs was observed in the first 2-4 weeks after starting 23 mg/d, after which the incidence of AEs was similar in both treatment groups. AEs usually associated with more advanced disease, such as neuropsychiatric symptoms [8], were not clinically significantly different between the 10 and 23 mg/d groups. There were no differences between groups in the incidence of clinically notable abnormal laboratory parameters or vital signs.

The 23 mg/d dose of donepezil was associated with a higher rate of AEs, particularly GI-related AEs (nausea, vomiting, diarrhea, and anorexia), than the 10 mg/d dose. The transient increase in these cholinergic AEs observed with initiation of the higher dose is consistent with the pattern previously seen in studies with a dose increase from 5 mg/d to 10 mg/d [9]. These results are in accord with other studies showing that AChEIs as a class are associated with cholinergic AEs and that the incidence and severity of these AEs increase when patients are titrated from a lower to a higher dose regimen [2,3]. However, previous studies did not specifically examine the temporal relationship between dose initiation and AEs.

During the first 2 weeks following upward dose titration, approximately 20% of patients reported a first episode of a GI-related AE. A less pronounced rise in GI-related AEs was also seen during this period in patients who continued treatment with donepezil 10 mg/d. This finding of early and transient increases in GI-related AEs following upward dose titration is consistent with those of previous studies in which GI AEs were associated with both treatment initiation and transitions from low-dose to higher dose treatment [3,4,7,9,10]. These events declined in frequency after 1 month of treatment, or about 2 weeks after steady-state donepezil levels had been reached [4,9,11]. Indeed, after 1 month, the proportion of patients reporting new occurrences of GI-related AEs in this trial fell to roughly 3% in both groups, and then remained low, steady, and similar between groups through Week 24. Discontinuations due to AEs also peaked during the first 2 weeks of the study in both treatment groups, with a higher incidence in the 23 mg/d group, and then the rate of discontinuations declined steadily thereafter. After 2 months of treatment, the rate of discontinuations remained low, steady, and similar between groups through Week 24.

Analysis of pooled data from 2 similarly conducted trials in mild to moderate AD [6,7] demonstrated that increasing donepezil dose from 5 mg/d to 10 mg/d led to a temporal pattern of AEs and discontinuations due to AEs similar to that observed with increasing from 10 mg/d to 23 mg/d. These data suggest that physicians and other prescribers may anticipate the same overall timing, type, and severity of AEs when changing patients' dose from 10 mg/d to 23 mg/d as has already been encountered when switching patients from 5 mg/d to 10 mg/d. No data are currently available to support an interim dose titration when increasing from donepezil 10 mg/d to 23 mg/d.

Rates of non-GI AEs with the 23 mg/d dose of donepezil were generally low but were greater than those seen with the 10 mg/d dose. Cardiac AEs were infrequent with both the 23 mg/d and 10 mg/d doses. Some studies have shown an increased risk of bradycardia and syncope in patients treated with AChEIs [12,13]. In the current study, the overall incidence of bradycardia was greater in the higher-dose group, and all 8 patients who discontinued the study due to bradycardia were taking 23 mg/d. However, bradycardia occurred in fewer than 3% of patients in the 23 mg/d group and was not commonly associated with other sequelae. Weight decrease as an AE was more common in the 23 mg/d group, though it occurred in fewer than 5% of patients, being mild to moderate in 45 patients and severe in only 1 patient.

Post hoc analyses to explore additional demographic factors showed that more women than men who increased dose to 23 mg/d had anorexia and weight loss. With respect to age, increased dose to 23 mg/d was associated with a higher incidence of fatigue, somnolence, and urinary incontinence in successively higher age groups. The higher rates of some common AEs observed in Asian/Pacific patients in both treatment groups may be related to lower mean weight in this patient subpopulation, as was found for the lower weight compared with the higher weight patients in general. A higher rate of AEs was observed in patients receiving memantine regardless of treatment group assignment, consistent with their more advanced stage of AD.

The study in which these data were obtained was a rigorously conducted global clinical trial involving a large number of patients with moderate to severe AD, of whom about one third were receiving concomitant memantine. Strengths of this study include the detailed temporal analysis, stratification by concomitant memantine use, and high completion rate in both groups. Limitations include lack of sufficient representation of Hispanic and black populations to assess potential race-related safety or tolerability issues, and a 24 week study duration.

## Conclusions

In this pivotal clinical study of donepezil 23 mg/d compared with donepezil 10 mg/d comprising more than 1400 patients with moderate to severe AD, donepezil 23 mg/d was shown to have acceptable tolerability and a favorable safety profile. Although a short-term increase in the rate of first reports of cholinergic-related GI AEs was observed during the initial titration phase, after the first few weeks of treatment the rates were similar in both the 23 mg/d and 10 mg/d treatment groups throughout the remainder of the study. The rate of serious AEs was low in patients treated with donepezil 23 mg/d and was similar to that seen with donepezil 10 mg/d. The 23 mg/d dose of donepezil was not associated with any new safety signals. The good safety and predictable tolerability profile for donepezil 23 mg/d supports its favorable risk/benefit ratio in patients with moderate to severe AD.

## Competing interests

Dr. Farlow is a paid consultant for Accera, Astellas, Bayer Bristol-Myers Squibb, Eisai Medical Research, GE Healthcare, Helicon, Medavante, Medivation, Inc., Merck and Co., Inc. Novartis Pharma, Pfizer, Prana Biotech, QR Pharma, Sanofi-aventis Groupe and Toyama Pharm.; is paid speaker for Eisai, Forest, Novartis, and Pfizer; and receives research support from Bristol-Myers Squibb, Elan, Eli Lilly and Co., Novartis Pharm., Pfizer, Octapharma, and Sonexa.

Dr. Felix Veloso has conducted clinical drug trials for and is in the speaker bureau of several pharmaceutical companies, including Pfizer, Lundbeck, Novartis, Janssen Ortho, Johnson & Johnson, Biogen Idec, sanofi aventis, Boehringer Ingelheim, Glaxo Smith Kline.

M. Moline, J. Yardley, E. Brand-Schieber, F. Bibbiani, H. Zou, T. Hsu, and A. Satlin are employees of Eisai Inc.

## Authors' contributions

MF participated in designing and conducting the study, and helped draft the manuscript. FV participated in monitoring the study and helped draft the manuscript. MM and TH conceived and designed the study, analyzed and interpreted the data, and helped draft the manuscript. JY, EB-S, FB, HZ, and AS analyzed and interpreted study data and helped to draft the manuscript. All authors read and approved the final manuscript.

## Pre-publication history

The pre-publication history for this paper can be accessed here:

http://www.biomedcentral.com/1471-2377/11/57/prepub
